# The fast–slow stretch-shortening cycle classification: provenance, transmission and evidential status of the 250 ms boundary

**DOI:** 10.3389/fphys.2026.1981174

**Published:** 2026-10-07

**Authors:** Chengzhi Li, Yi Yang

**Affiliations:** College of Physical Education, Qingdao Hengxing University of Science and Technology, Qingdao, China

**Keywords:** achilles tendon, classification, ground contact time, impulse, plyometric training, reactive strength index, rehabilitation, stretch-shortening cycle

## Abstract

Ground contact time is widely used to classify plyometric exercises as fast (< approximately 250 ms) or slow (> approximately 250 ms) stretch-shortening cycle (SSC) actions, shaping drill selection, progression and study design in training and rehabilitation. A categorical threshold is informative only if it marks a reproducible change in mechanism, in outcome, or in decision value, defined here as its capacity to change a prescription decision such as drill selection or progression. We examine the source of the 250 ms boundary, its original criteria, the evidence for a mechanistic breakpoint, what contact time indexes mechanically, the determinants of category membership, and the consequences for prescription and synthesis. In the source, long and short SSC actions were defined using two joint conditions, movement duration and angular displacement at the hip, knee and ankle, and the short category was bounded between 100 and 250 ms. This definition was subsequently reduced in much of the research literature to a one-sided ground-contact-time threshold without documented validation, whereas both criteria were often retained in applied accounts. The only direct test, in 46 recreationally active men, did not reproduce 250 ms and identified Achilles tendon length as the strongest discriminator of contact-time groups. Existing mechanistic evidence distinguishes slow from fast tasks but does not identify a discontinuity at 250 ms. *In vivo* and impulse–momentum evidence shows instead that contact time indexes how a required impulse is delivered and how length change is partitioned between tendon and muscle, and that both vary continuously with task constraints. In parallel, common intensity metrics rank drills differently, and fast SSC drills have contact times two to three times longer than a sprint contact. In rehabilitation the boundary is used as an injury-dependent progression gate, and meta-analyses have not been stratified by the criterion used to assign categories. These findings do not establish that 250 ms is useless; they show that it should be treated as an unvalidated operational heuristic rather than a biologically privileged boundary. Its value should be tested prospectively by asking whether category membership predicts training response once tendon geometry, task and measurement are accounted for.

## 1 Introduction

Ground contact time is often used to convert a continuum of plyometric actions into two categories: fast stretch-shortening cycle (SSC) actions below approximately 250 ms and slow SSC actions above it. The distinction informs drill selection, exercise progression and study design. The depth jump was formalized as a training method before this binary rule became established (Bobbert, 1990), and plyometric intensity has since been operationalized using electromyographic activity (Ebben et al., 2008), kinetic characteristics (Ebben et al., 2011) and drop height (Lees and Fahmi, 1994). These measures grade exercises along continua and can yield different orderings. The practical question is therefore not simply whether contact time is measurable, but whether a 250 ms threshold adds information beyond other descriptors. This matters because coaches choose among drills with distinct contact demands, from single box jumps to repeated hurdle hops, and their programming choices have been documented directly (Loturco et al., 2023).

The distinction remains active rather than merely historical. In studies published in 2025 and 2026, jump actions continue to be grouped as slow versus fast (McGarrigal et al., 2025), long versus short (Freitas et al., 2026), or distinct SSC types (Cavar et al., 2026). At the same time, vertical-jump adaptations have recently been described across an SSC continuum rather than between two classes (Rodríguez-Fernández et al., 2026). The coexistence of a binary classification and continuum language makes the underlying construct more important to examine: what, exactly, does the boundary separate?

Whether 250 ms identifies reproducible groups has been tested directly in only one study. Ünver et al. (2024) noted that no explanation of how the value had been derived, or of which variables determine it, was available in the literature. In 46 recreationally active men, data-derived cut-points occurred at approximately 188 and 222 ms, producing three groups rather than two; Achilles tendon length was the strongest discriminator. These findings raise two separate questions: whether 250 ms is the appropriate cut-point, and whether category membership reflects the exercise or the athlete. Earlier evidence that musculotendinous stiffness and tendon elastic properties influence jump performance makes the second question plausible (Walshe and Wilson, 1997; Kubo et al., 1999). More broadly, commonly used measures of lower-body SSC utilization do not agree with one another (Suchomel et al., 2016).

Three developments make this re-evaluation timely. First, in a systematic review of 184 studies and 209 neuromuscular profiling methods, terminology, definitions and calculations were found to be inconsistent (Chua et al., 2026), raising a general concern about whether labels correspond to what is measured. Second, debate about whether slow and fast SSC actions involve distinct neuromechanical control has been reopened in an article, a letter and a reply (Rissmann et al., 2025; Taube et al., 2025; Hahn et al., 2025). The SSC has served for decades as a model of muscle function and fatigue (Komi, 2000; Nicol et al., 2006), but that exchange concerns mechanisms rather than the origin, predictive validity or practical function of the 250 ms boundary. Third, Xu et al. (2025), in an applied review, described the threshold as arbitrary and proposed a narrower 65–175 ms criterion. The central issue, however, may lie not only in the value chosen but also in what the classification is expected to predict.

The rationale for re-examining the boundary is therefore conceptual as well as empirical. A categorical threshold is not a neutral descriptor: it defines what counts as fast SSC training in a trial, decides which interventions are grouped together in a synthesis, and is a gate in progression schemes for athletes and for patients returning from injury. If the quantity on which it is applied depends on the athlete as much as on the exercise, the category confounds the stimulus with the person who receives it. Empirically, the three developments above show that the number is being re-derived without agreement on what a threshold should predict. One study derived cut-points near 188 and 222 ms, one review proposed 65–175 ms, and one side of the 2025 exchange denied that any physiologically meaningful division exists. A further number would not resolve this disagreement. What is missing is a statement of the conditions under which any cut-point would be informative, and an assessment of whether the existing evidence meets them. The relevant evidence is archival, mechanistic, cross-sectional and meta-analytic, and it cannot be pooled or graded by a single tool. A critical narrative review is the appropriate design for such a question: its purpose is to test the coherence of an argument and to specify what a subsequent systematic review or prospective study should measure (Greenhalgh et al., 2018).

The question has direct practical consequences, so the argument is framed for two settings. In performance training, fast SSC drills such as drop jumps and hurdle hops are prescribed to transfer to sprinting and jumping. In rehabilitation, the same drills are reintroduced after injury under different constraints. New cut-points would change little for practitioners if categories continued to be assigned by drill name. What would change practice is recognizing what contact time indexes: the way in which a required impulse is distributed in time and, with it, the partition of length change between tendon and muscle and the loading rate imposed on tissue. On that reading, the same drill can deliver a fast stimulus to one athlete and a slow stimulus to another. A hurdle hop performed on two legs and on one leg can fall on opposite sides of the line (Cappa and Behm, 2011). The boundary used to clear a patient for reactive loading answers a different question from the boundary used to select a drill for a sprinter. Making these distinctions explicit, rather than replacing one number with another, is the practical contribution sought here.

In this critical review, the fast–slow SSC classification is evaluated through four linked questions. How did the boundary and its original criteria enter the literature? Does evidence support a mechanistic breakpoint at 250 ms, and what does contact time index at the level of muscle–tendon interaction and impulse? What determines category membership for a given athlete and task? Does the classification improve prescription, in performance training and in rehabilitation, or evidence synthesis? Whether plyometric training is effective is not reassessed, and effect sizes are not pooled. Tendon properties are considered as potential determinants of category membership, not as training targets. Evidence that contact conditions influence neuromuscular adaptation (Taube et al., 2012a) is used to ask what the classification tracks, not to rank one category above another.

## 2 Methods

This critical review was informed by targeted searches of PubMed/Medline, SPORTDiscus, and Web of Science (last search: 2 September 2026) using classification- and measurement-specific terms (e.g., stretch-shortening cycle, ground contact time, fast SSC, slow SSC, drop jump, reactive strength index, plyometric), with no date limit. Evidence was selected primarily for direct bearing on the four questions set out in the Introduction: the provenance of the boundary, mechanistic evidence for a breakpoint and what contact time indexes, determinants of category membership, and consequences for prescription and evidence synthesis. Additional contemporary sources were identified through backward and forward citation tracking of key papers, in particular Schmidtbleicher (1992) and Flanagan and Comyns (2008). Studies were excluded when they neither measured contact time nor reported the criterion by which exercises were assigned to SSC categories. During revision, supplementary targeted searches (September 2026) were made for *in vivo* muscle–tendon behavior during hopping and drop jumping, ground contact times in sprinting and hurdle jumping, and plyometric progression in rehabilitation. These sources frame the applied sections and were selected for direct relevance rather than screened by the criteria above. This process yielded the 106 sources cited in the review.

## 3 How the 250 ms boundary emerged

The boundary can be traced to Dietmar Schmidtbleicher’s chapter on training for power events in the International Olympic Committee volume edited by Paavo Komi (Schmidtbleicher, 1992). Several points that are obscured in later accounts are settled in that chapter: each category is defined using two conditions, the labels differ from current terminology, and the short category is bounded at both ends. No evidence for the numerical limits is presented, however. The limits are attributed to an earlier German-language publication by the same author (Schmidtbleicher, 1986), which could not be obtained for this review. **Figure 1** traces the subsequent transmission of the criteria.

**Figure 1:**
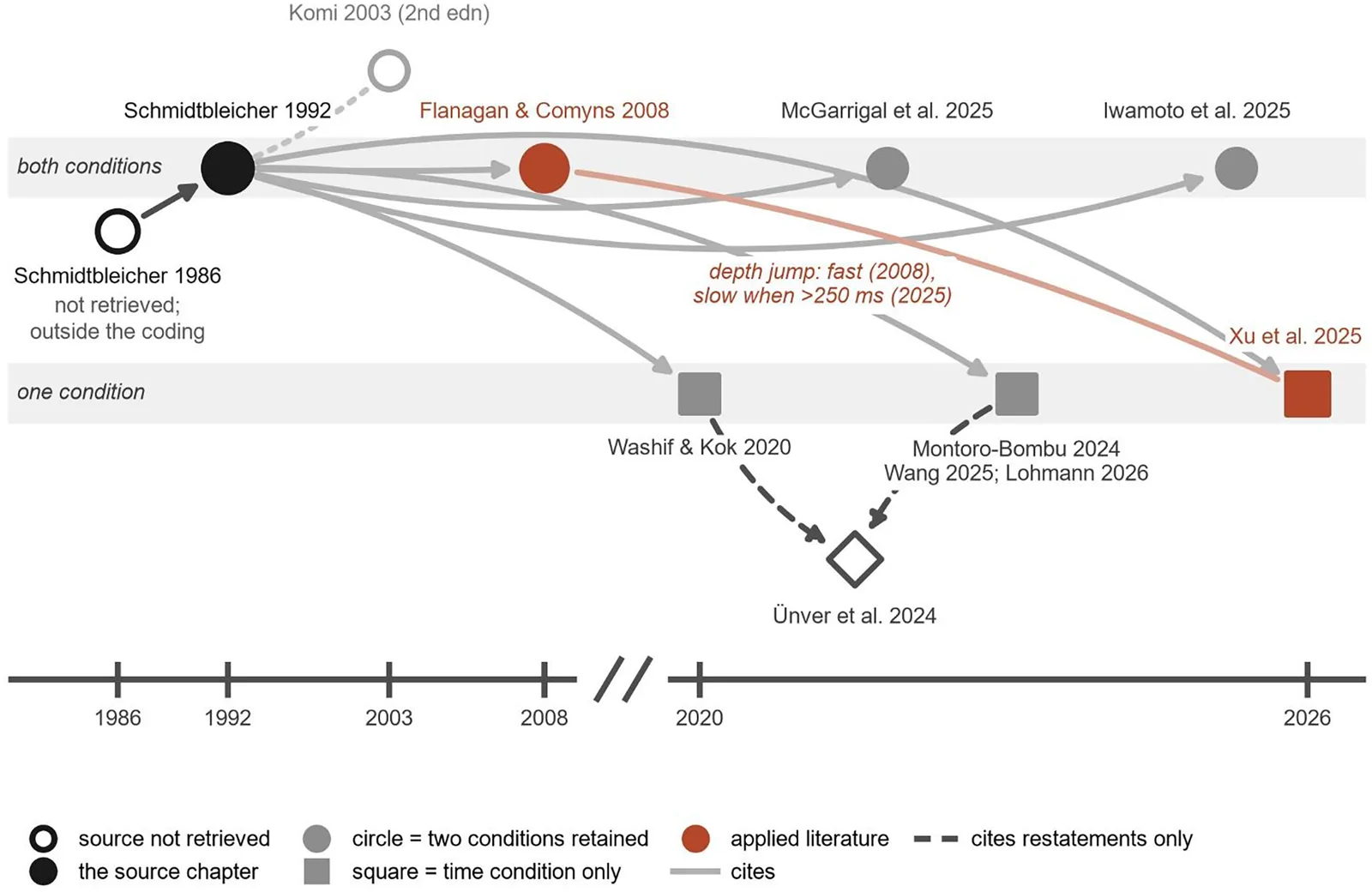
Transmission of the fast–slow criterion from its source into current use. Horizontal position denotes publication year; vertical position is ordinal with three levels, according to whether an account retains both of the original defining conditions, one of them, or states neither. The dashed, unfilled node for Schmidtbleicher (1986) lies outside this coding because the report was catalogued but not obtained. Solid arrows show citation direction, not influence or causation. Recent accounts are spaced for legibility. Accounts shown and the criterion stated in each. They were selected to represent every variant form identified in the screened corpus and to span the period from the primary sources to current use. Schmidtbleicher (1986): the report to which the 1992 chapter attributes its numerical limits; shown unfilled with a dashed outline and outside the retention coding because it was catalogued but not obtained for this review and is absent from the reference lists of accounts that apply the threshold. Schmidtbleicher (1992): two joint conditions, with the short category defined as a 100–250 ms interval and the measured quantity described as duration. Komi (2003), second edition: the Schmidtbleicher chapter was not retained. Flanagan and Comyns (2008): both conditions and the individual prescription criterion were retained, with the quantity described as contraction time. Research, 2020 (Washif and Kok, 2020): the threshold was reported as 0.250 s. Research, 2025 (McGarrigal et al., 2025; Iwamoto et al., 2025): one account combined ground contact time with angular displacement; the other retained both conditions, the label long and the term movement time. Research, 2024–2026 (Montoro-Bombú et al., 2024; Wang et al., 2025; Lohmann et al., 2026): ground contact time alone. Xu et al. (2025): the threshold was described as arbitrary and a 65–175 ms criterion proposed. Ünver et al. (2024): the only direct validation study; its reference list included restatements but neither the 1992 chapter nor the 1986 report. The curved connector links two uses of depth jump: as the typical fast-category exercise in 2008 and as an exercise placed beyond the 250 ms boundary in 2025, both in the same journal. Bibliographic details also drifted. In the 46 reference entries checked, the chapter’s actual span (381–396) never appears: 27 give 381–395, one gives 381–385, seven give no pages, and 11 give 169–179, the location of Komi’s separate chapter on the SSC (Sect. 3.3).

### 3.1 The definition in the 1992 chapter

In the chapter, two SSC types are defined through the conjunction of movement duration and joint displacement. A long cycle involves large angular displacements at the hip, knee and ankle and lasts more than 250 ms; examples include jumps used to throw in basketball and to block in volleyball. A short cycle involves small displacements at the same joints and lasts 100–250 ms; examples include the ground-contact phases of sprinting, high jumping and long jumping (Schmidtbleicher, 1992). This differs from current usage in three consequential ways. The criteria are joint rather than alternative, the short category is a bounded interval rather than every value below a ceiling, and the measured quantity is described as duration rather than ground contact time. The drop jump, now commonly treated as the fast-cycle archetype, is not used to define either category, although it appears elsewhere in the chapter. The numerical limits are attributed to the earlier German-language report (Schmidtbleicher, 1986), and no supporting data are reproduced in the 1992 chapter.

The use of 250 ms and of labels within the chapter is also internally inconsistent. Earlier, movements lasting 250 ms or less are associated with rate of force development, whereas longer movements are associated with maximal strength (Schmidtbleicher, 1992). That claim is not connected to the later SSC classification, and the subsequent discussion of training methods refers to fast and slow cycles without reconciling those terms with the long and short categories used in the definition. Terminological instability therefore begins in the source itself, not only in later citations.

### 3.2 How the definition changed in transmission

As the definition travelled, it became simpler and more categorical. Long and short became slow and fast, terms already used in the physiological literature on muscle types (Bosco et al., 1982). The lower bound of 100 ms disappeared, and duration became ground contact time. The drop jump replaced sprinting and jumping contacts as the reference case, while individually adjusted drop height gave way to population-level heights. In the source chapter, drop height was set for each athlete so that the heel did not reach the ground, and the training effect was considered doubtful when contact was either too short or too long (Schmidtbleicher, 1992). In contemporary research, an absolute threshold is more often applied and drop height is treated as a population-level prescription variable.

These changes were not uniform. In the applied account that most directly operationalized contact time for coaches, both defining conditions were retained, the quantity was described as contraction time and the individual heel-contact criterion was preserved (Flanagan and Comyns, 2008). The simplification is more evident in the research citation chain. Both conditions, the label long and the term movement time are retained in one recent account (Iwamoto et al., 2025). A 0.250 s threshold is reported in others (Washif and Kok, 2020), and in one the threshold is stated in units of length: “Based on ground contact time, SSC is classified into slow (>250 mm) and fast (<250 mm) types” (Wang et al., 2025). Removing a criterion is not necessarily erroneous, but it requires evidence that the omitted information does not improve classification. No such justification was identified. The removed joint-displacement criterion is also the one most likely to distinguish a long contact caused by movement strategy from one associated with the anatomical factors discussed later in this review. The same point has since been made from the applied side: an athlete using a stiff rebounding strategy with minimal joint displacement but a contact of 251 ms would still exhibit ballistic characteristics despite exceeding the threshold (Xu et al., 2025).

The reference exercise also shifted. In the 2008 applied account, the depth jump was described as a typical fast SSC action (Flanagan and Comyns, 2008). By contrast, in a 2025 review in the same journal, the depth jump was placed in the slow category when contact exceeded 250 ms and drop jump was reserved for the fast category (Xu et al., 2025). In neither account is the terminology marked as a departure. Thus, both the criterion and the exemplar now vary across sources.

### 3.3 The current citation chain

Current publications carry at least two versions of the construct. In some, SSC type is defined using ground contact time together with angular displacement at the hip, knee and ankle (McGarrigal et al., 2025), whereas in others ground contact time is used alone (Wang et al., 2025; Montoro-Bombú et al., 2024; Lohmann et al., 2026). Bibliographic drift accompanies this conceptual drift. In the copy of the first edition consulted for this review (Blackwell Scientific Publications, ^©^ 1992 International Olympic Committee, ISBN 0-632-03031-3), the chapter occupies pages 381–396. Of the 46 citing works whose reference entries for the chapter could be extracted and checked in a full-text screen of the PubMed Central open-access corpus (90 works identified as citing the chapter or stating the threshold, 81 with retrievable full text; last search: 2 September 2026), none gives this span. Twenty-seven give 381–395, the range printed in the second edition’s own reference list; one gives 381–385 (Snyder et al., 2018); seven give no pages; and 11, among them Montoro-Bombú et al. (2024); Kozinc et al. (2021) and Barry et al. (2016), give 169–179, the opening pages of Komi’s separate chapter on the SSC in the same volume (Komi, 1992). In all 11, and only these 11, the book is titled with the Encyclopaedia of Sports Medicine series form, so the misattributed range travels with a particular citation format rather than arising independently. These counts describe the open-access works examined, not the citing literature as a whole. The Schmidtbleicher chapter was not retained in the second edition; there, Komi used fast and slow descriptively without specifying a threshold (Komi, 2003).

In the direct validation study, intermediate accounts rather than the primary source were relied upon: neither the 1992 chapter nor the 1986 report appears in its reference list (Ünver et al., 2024). The statement by its authors that the origin of 250 ms could not be recovered is therefore accurate for the English-language citation chain they examined. The problem is not that the 1992 source is unavailable, but that the chain commonly stops at restatements of it. The earlier German-language report exists and is catalogued: searches of the German National Library and the K10plus union catalogues (2 September 2026) identify it as the documentation of a December 1985 Cologne workshop, published in 1986 by the Bundesinstitut für Sportwissenschaft (ISBN 3-89001-069-5). No digitized copy was located; the printed volume is held in German collections but was not obtained for this review: interlibrary loan could not be completed within the revision timeframe, and neither the publisher nor the author’s institution was contacted. Its contents therefore cannot be inferred here, and the present argument does not require such inference. The narrower conclusion is that the evidence supporting the numerical limits has not been accessible to, or reproduced in, the literature that routinely applies them.

## 4 What would make 250 ms a meaningful boundary

A practical cut-point need not correspond to a literal discontinuity in biology. It should, however, add reproducible information: a change in mechanism, an outcome difference, or a decision rule that predicts who responds to which stimulus. Without one of these forms of validity, 250 ms is a convenient label rather than an explanatory category. The question addressed in this section is therefore whether the proposed mechanisms identify a privileged boundary near 250 ms. The evidence is limited and comes largely from single muscles, controlled preparations and specific tasks, so each mechanism is considered with its experimental scope intact.

### 4.1 Proposed mechanisms and their evidence

A preceding stretch can augment muscle work, as documented since 1968 (Cavagna et al., 1968), and slow and fast tasks clearly differ at the ends of the movement spectrum. Those differences have recently been attributed to opposing motor-control patterns: slower eccentric actions involve fiber lengthening and spinal inhibition, whereas faster stretching involves muscle activation, spinal excitation and tendon lengthening (Taube et al., 2025). A hop and a countermovement jump are therefore not equivalent. The unresolved question is whether the transition between them occurs at a reproducible value, and specifically at 250 ms.

Several non-exclusive mechanisms could contribute to SSC performance, but the variables implicated in them vary continuously. Muscle–tendon unit and fascicle decoupling is one candidate: preceding stretch increased joint torque and work, muscle–tendon unit force and work, and fascicle force by 12–22%, while fascicle work decreased by approximately 35% (Holzer et al., 2024). Fascicle behavior also changed with loading rather than at a reported threshold. Across increasing drop heights, medial gastrocnemius fascicles shifted from shortening during braking to isometric or lengthening behavior, whereas soleus fascicles lengthened before shortening throughout (Sousa et al., 2007). Highly active cycles in the medial gastrocnemius may also be uncommon even when joint- and tendon-level cycles are prominent (Aeles and Vanwanseele, 2019). Other candidates include history-dependent force production (Hahn et al., 2023), titin-related mechanisms in skinned fibers (Tomalka et al., 2024), and intensity-dependent effects (Held et al., 2023). Height, velocity and intensity are implicated in these studies, but a breakpoint at 250 ms is identified in none of them. The same pattern holds across the wider mechanistic literature. Pre-activation and reflex contributions vary with drop height, movement speed and surface (Taube et al., 2012a, 2012; Márquez et al., 2014), and tendon buffering of fascicle strain has been documented across tasks of different speeds (Sousa et al., 2007; Aeles and Vanwanseele, 2019); history-dependent and titin-based force mechanisms have been characterized in reduced preparations (Hahn et al., 2023; Tomalka et al., 2024). What these lines of work share is their design: conditions are drawn from the ends of the task range, and contact times have not been sampled densely around 250 ms in any of them to test for a transition there. The absence of a demonstrated breakpoint is thus a property of the whole mechanistic corpus, not of the 2025 exchange alone.

### 4.2 Current disagreement about mechanism

The contribution of neural and mechanical mechanisms remains disputed. In electromyographically matched, submaximal plantar-flexor contractions performed prone, a 12% SSC effect occurred without detectable modulation of cortical or spinal excitability or stretch-reflex activity, leading the authors to favour a mechanical origin (Rissmann et al., 2025). The generalizability of that conclusion was questioned in a subsequent letter (Taube et al., 2025). The stretch velocity of 40 degrees per second was far below the 280–700 degrees per second reported for hopping and drop jumps, so the protocol was interpreted as a slow SSC task that could not represent faster actions. This interpretation is consistent with earlier accounts of joint feedforward and feedback control (Taube et al., 2012b). In their reply, Hahn et al. (2025) accepted the limited task generalizability but rejected the proposed binary division. They argued that muscle–tendon decoupling weakens the link between joint and fascicle velocities, that reflex interpretation cannot rest on contact time alone, and that no physiologically meaningful slow–fast threshold has been demonstrated.

### 4.3 What the disagreement establishes

250 ms is not established as a biologically privileged value by either side of this exchange. If slow and fast tasks involve different motor-control patterns, the cited velocities demonstrate separation at the ends of the range but do not locate the transition. If no physiologically meaningful threshold exists, contact time cannot supply one by itself. A discontinuity at another value remains possible, but it has not been observed directly. This is the least secure evidence stream in the review. The historical argument rests on documents that can be compared, and the determinants of category membership are supported by measurements in identifiable cohorts. By contrast, the mechanistic argument rests on a small number of muscle- and task-specific studies. The defensible conclusion is therefore that a discontinuity has not been demonstrated, not that it has been ruled out.

This limitation does not make contact time uninformative. Contact duration covaries with mechanically and physiologically relevant quantities, and drop height can alter both contact time and the direction of neuromuscular adaptation (Taube et al., 2012a). Force–time traces also contain structure that a single duration cannot capture (Peng et al., 2024). The key distinction is between a useful descriptor and a validated classifier. Contact time may describe an action well without proving that 250 ms separates two mechanisms or two training responses. What it does describe is examined next.

## 5 What contact time indexes: muscle–tendon interaction and impulse

If no discontinuity has been demonstrated, the next question is what contact time does track. Two bodies of evidence answer it more precisely than the fast–slow label: *in vivo* studies of how length change is partitioned between tendon and muscle during bouncing tasks, and the impulse–momentum relation that links contact time, peak force and take-off velocity. Both show that contact time indexes the way in which a mechanical demand is delivered rather than the size of the demand, and both show that this varies continuously with task constraints. The evidence is set out here because it changes what the classification can be expected to predict in the applied sections that follow.

### 5.1 Task constraints set the partition between tendon and muscle

During bouncing tasks the muscle–tendon unit lengthens and shortens, but the fascicles and the tendon need not do so in proportion. During one-legged hopping at approximately 2 Hz, the Achilles tendon accounted for over 80% of the length change of the muscle–tendon unit throughout most of the hopping cycle (Lichtwark and Wilson, 2005). Peak tendon strain averaged 8.3%, hysteresis was 17–35%, and the 38 J of energy returned by the tendon corresponded to 16% of the mechanical work of the hop (Lichtwark and Wilson, 2005). Hopping is preferentially performed at about 2.2 Hz, a frequency at which the body behaves as a simple spring–mass system; spring-like behavior persists at higher frequencies but diminishes below the preferred frequency (Farley et al., 1991). In that study contact time itself crossed the boundary. At the preferred frequency, contact lasted 0.302 ± 0.008 s during normal hopping but 0.203 ± 0.005 s when the same subjects hopped for maximum height. At the highest attainable frequency of 3.6 Hz, the two values fell to 0.204 ± 0.004 and 0.173 ± 0.004 s (Farley et al., 1991). Normal hopping at about 2 Hz has contacts of about 0.3 s, which a 250 ms rule would classify as slow, yet the tendon takes up most of the length change. Instruction alone moves the same task to the other side of the line.

Across hopping at 2, 2.5, 3 and 3.5 Hz, the contribution of tendon length change to muscle–tendon unit length change rose from 86% at 2 Hz to 94% at 3 Hz and then fell to 60% at 3.5 Hz (Monte et al., 2021). Tendon gearing, the ratio of muscle–tendon unit length change to muscle-belly length change, followed the same course (5.5, 6.6 and 4.6). The mechanical power released by the tendon and the apparent efficiency of the movement (0.44, 0.51, 0.52 and 0.40) were highest at 2.5–3 Hz, where the muscle belly contracted most nearly isometrically (Monte et al., 2021). Maximum knee flexion fell over the same range from 51° to 29° (Monte et al., 2021), so joint displacement and contact time covaried in the manner assumed by the original two-condition definition, but continuously. The frequency of a bouncing task therefore determines how much of the stretch is taken by the tendon and how much by the muscle. The relation is neither monotonic nor located at a single frequency.

The same graded behavior appears when task intensity, rather than frequency, is manipulated. Across increasing drop heights, medial gastrocnemius fascicles shifted from shortening during braking to isometric or lengthening behavior, whereas soleus fascicles lengthened before shortening at every height (Sousa et al., 2007). Highly active fascicle stretch–shortening in the medial gastrocnemius can be uncommon even when cycles at the joint and tendon level are prominent (Aeles and Vanwanseele, 2019). Preceding stretch increased fascicle force while decreasing fascicle work, the signature of decoupling between the fascicles and the muscle–tendon unit (Holzer et al., 2024). The partition is also athlete-dependent: vertical leg stiffness during drop jumps differed by approximately 70% between sprinters and endurance athletes (Harrison et al., 2004), and tendon geometry and material properties vary with loading history (Section 6.1). Contact time covaries with the tendon–muscle partition, which is what makes it informative, but the covariation is continuous and depends on frequency, height, instruction and the tissue properties of the athlete. Nothing in this evidence marks 250 ms as the point at which tendon-dominated length change gives way to muscle-dominated length change.

### 5.2 Impulse, peak force and contact time

The second body of evidence is mechanical rather than physiological. For a jump from a given drop height to a given take-off velocity, the net vertical impulse generated during ground contact is fixed by the change in momentum of the body. It equals body mass multiplied by the sum of the landing and take-off speeds, and it does not depend on how long contact lasts. Contact time determines only how that impulse is distributed. A shorter contact requires a higher mean net force and, in practice, a higher peak force and loading rate, whereas a longer contact allows the same impulse to be delivered at lower force. This is the mechanical basis of the technical distinction between a drop jump and a depth jump (Pedley et al., 2017). In the drop jump, leg flexion is restricted and downward velocity is reversed as quickly as possible; in the depth jump, flexion is unrestricted and jump height is maximized. Instructing athletes to minimize ground contact reduces jump height and increases peak vertical ground reaction force and loading rate (Pedley et al., 2017). Maintaining jump height while shortening contact requires greater force in a shorter time, because the vertical impulse needed for the same take-off velocity is unchanged (Pedley et al., 2017). In a comparison summarized by Xu et al. (2025), the depth jump from a 0.45 m box lasted 420 ± 70 ms and the drop jump 180 ± 30 ms, a difference attributed to the greater joint displacement of the former. Two jumps from the same height that reach the same take-off velocity share an identical net impulse whatever their contact times. What differs is the task demand, the peak force and the tissues that absorb it; as Section 5.1 indicates, a larger share of the length change is taken up by the tendon in the shorter contact. The same distinction underlies the placement of the depth jump beyond 250 ms and the reservation of the drop jump for the fast category in recent applied work (Xu et al., 2025).

Kinetic data show that the classifier and the mechanical demand do not move together in a single direction. Across drop heights of 0.2, 0.4 and 0.6 m, force and time variables increased together, so that a higher box lengthened contact while raising force (Ball et al., 2010). Across drills, peak force, time to take-off and landing rate of force development ranked exercises differently (Ebben et al., 2011). Contact time, impulse and peak force are therefore three separate descriptors, and a classification based on the first cannot stand in for the other two. Framed in this way, plyometric intensity is better expressed by the impulse required and the rate at which it is delivered than by the side of a 250 ms line on which a drill falls. The classification remains useful as a coarse index of loading rate, but only when the impulse it distributes is also known.

### 5.3 What follows for the classification

Three consequences follow. First, where the boundary is informative at all, it marks a region of high loading rate in which, up to a point, the tendon takes a larger share of the length change (Section 5.1). It does not mark a change in the amount of mechanical work performed. Second, because frequency, height, instruction and tissue properties all move a task along this continuum, category membership is a property of the athlete performing the task under particular constraints (Section 6). Third, the practical value of the boundary depends on the setting (Section 7). In performance training the question is whether a drill reproduces the impulse and loading rate of the target action. In rehabilitation it is whether a healing tissue can tolerate the loading rate that a short contact imposes.

## 6 Category membership depends on athlete and task

The findings of the study in which 250 ms was not reproduced also bear on how category membership should be interpreted. In that cohort, Achilles tendon length was the strongest discriminator of the contact-time groups: shorter tendons were associated with shorter contacts and longer tendons with longer contacts (Ünver et al., 2024). A single study cannot establish a universal determinant, but the result shows that a threshold may classify the athlete as well as the exercise. The evidence can be organised by timescale. Tendon geometry changes little, tendon material properties adapt over weeks, and leg stiffness, technique and surface can change contact behaviour within a session. A fixed threshold applied across these levels risks combining stable anatomy with modifiable task conditions.

### 6.1 Anatomy may influence membership without determining performance

Tendon geometry and mechanics vary substantially between athletes and are shaped by long-term loading history. Musculotendinous stiffness was associated with drop-jump performance in 1997 (Walshe and Wilson, 1997), and tendon elastic properties with jump performance in 1999 (Kubo et al., 1999). In a direct group comparison, sprinters and endurance athletes differed by approximately 70% in average vertical leg stiffness during drop jumps, 5.86 versus 3.38 kN·m^-^¹ (Harrison et al., 2004). Habitual movement patterns also covary with tissue properties. Forefoot-strike runners show higher gastrocnemius shear elastic moduli, passive ankle stiffness and Achilles tendon cross-sectional area than non-forefoot-strike runners (Hirono et al., 2022). Dancers show greater Achilles tendon elongation, hop height and relative peak power than runners or untrained controls (Rice et al., 2021). None of these findings shows that anatomy alone determines contact time. Together, however, they make between-athlete differences expected even when the nominal drill and instruction are identical, because the partition of length change between tendon and muscle depends on tendon compliance and gearing (Section 5.1).

The claim that tendon morphology influences category membership is distinct from the claim that morphology predicts performance. Evidence for the latter is limited and often negative. In 48 well-trained sprinters, Achilles tendon length and cross-sectional area were not associated with personal-best 100 m time, whether expressed absolutely or normalized to shank length (Tomita et al., 2021). A longer Achilles tendon moment arm was not associated with better repetitive hopping; maximal isometric plantar-flexion force and muscle size were also unrelated to hopping performance (Kovács et al., 2023). Similarly, sprinters and endurance athletes differed in leg stiffness and jump outcomes, but not in prestretch augmentation, the performance benefit attributed to the SSC (Harrison et al., 2004; Bosco and Komi, 1979). These null associations concern distal outcomes and should not be overgeneralized, but they show that the anatomy linked to contact-time grouping need not map directly onto performance.

Taken together, the evidence supports a bounded interpretation. Achilles tendon length separated contact-time groups in one cohort (Ünver et al., 2024), whereas related anatomical measures have not consistently predicted sprint or hopping performance (Tomita et al., 2021; Kovács et al., 2023). Category membership may therefore encode anatomy and loading history without independently identifying stimulus quality. This is a hypothesis generated by the combined evidence, not a settled mechanism, and it requires prospective testing in the same athletes.

### 6.2 Context changes contact time across multiple timescales

Contact time, and therefore category membership near the threshold, is context-sensitive. Across maturation, prepubescent boys showed longer total contact times, lower reactive strength index values and less knee flexion at touchdown than men during individually scaled drop jumps (Bassa et al., 2022). Yet force–time classification within youth still identified 17.8% of post-peak-height-velocity participants as having poor SSC function (Kumar et al., 2024), showing that maturity does not determine profile on its own. Within a session, exhaustive rebound jumping reduced leg stiffness by 27 ± 12%, whereas preceding maximal jumps did not (Kuitunen et al., 2007). Technique changed the same athlete’s response: bounce drop jumps produced shorter contact, amortization and take-off phases than countermovement drop jumps at 30, 45 and 60 cm (Bobbert et al., 1987a; Struzik et al., 2016). Surface also redistributed soleus reflex excitability (Márquez et al., 2014) and produced surface-specific adaptations over eight weeks (Ahmadi et al., 2021). Thus, age, fatigue, technique and environment can move the measured quantity without representing the same biological process.

Training may alter tendon behavior without changing the geometry implicated in group assignment. After 14 weeks of plyometric training, Achilles tendon dissipation decreased by 35.0%, cross-sectional area did not change, and stiffness showed only an upward trend (Fouré et al., 2010). The authors interpreted this as a qualitative change in tendon tissue rather than a geometric change. This finding does not prove that contact-time membership is fixed, but it illustrates the mismatch in timescale: the candidate anatomical discriminator may remain stable while performance-relevant material properties adapt.

For practice, this evidence supports caution rather than a new rule. The same prescribed drill can produce different contact times across athletes, and the difference may reflect anatomy, maturation, fatigue, technique, surface or measurement; a single contact-time value cannot identify which source is responsible. The only direct validation study included 46 recreationally active men (Ünver et al., 2024); women, youth and highly trained athletes remain untested, several supporting studies involved fewer than 20 participants, and men are over-represented across the evidence base. The direction of context effects is therefore better supported than their magnitude or generalizability. Until anatomy and training response are measured prospectively in the same participants, category membership should not be assumed to provide a transferable unit of exercise intensity.

## 7 Consequences for prescription in performance training and rehabilitation

The classification is commonly asked to perform three applied functions: rank drills by intensity, calibrate the outcome used to judge training, and guide exercise selection. Each function requires a different form of validity. The question is not whether contact time contributes useful information, but whether the fast–slow label improves a decision beyond the measurements from which it is derived. This distinction keeps construct validity separate from the well-supported effectiveness of plyometric training. The two settings in which the label is applied are considered in turn, because the decision it is asked to support differs between them. In performance training the boundary is used to match a drill to a target action. In rehabilitation it is used to decide when a healing tissue may be exposed to reactive loading.

### 7.1 Competing intensity rankings

Plyometric intensity is operationalized in several ways, and the resulting rankings are not interchangeable (**Table 1**). Electromyographic comparisons can place drills conventionally described as high intensity, including the depth jump, below drills described as lower intensity (Ebben et al., 2008). In kinetic approaches, peak vertical ground reaction force at take-off, time to take-off, flight time, jump height, peak power, landing rate of force development and landing peak force are used; these variables need not order exercises identically (Ebben et al., 2011). Drop height is widely used as a proxy, but its effects are not consistently monotonic. Heel contact was found at the greatest tested height in early work, and a limit to the drop was recommended (Bobbert et al., 1987b). Across 20–90 cm, jump performance and neuromuscular markers changed without a clear dose–response pattern, and individual muscles showed increased, decreased or unchanged activity as height rose (Andrade et al., 2020). A higher box therefore does not guarantee a larger or more relevant stimulus.

**Table 1:** Parallel operationalisations of plyometric intensity and why their rankings diverge.

Operationalisation	What is measured	Common interpretation	Why it may conflict with other measures	Source
Ground contact time	Duration of foot contact	Applies a binary fast–slow label in common use	No evidence identifies 250 ms as a reproducible mechanistic or response breakpoint, and the same impulse can be delivered over different contact times (Sect. 5.2)	This review
Electromyographic activity	Motor unit recruitment across drills	Can place the depth jump below drills conventionally labelled lower intensity	Electromyographic rankings can differ from conventional drill hierarchies	(Ebben et al., 2008)
Kinetic quantification	Peak vertical ground reaction force at take-off, time to take-off, flight time, jump height, peak power, landing rate of force development, landing peak force	Multiple kinetic variables can generate different rankings	No single ranking follows from the kinetic data alone	(Ebben et al., 2011)
Drop height	Height of the platform	Often treated as a monotonic increase in loading	Responses can be non-monotonic, and greater height can lengthen contact time	(Lees and Fahmi, 1994; Bobbert et al., 1987b; Andrade et al., 2020; Ball et al., 2010)
Reactive strength index	Jump height divided by contact time	Discriminates among drills and supports individual calibration	When used for both calibration and outcome assessment, it cannot independently validate the system	(Ebben and Petushek, 2010; McMahon et al., 2021)

Drop-height prescriptions are re-derived in each population and use non-equivalent units: below 75% of maximum vertical-jump height in Division III athletes, whereas 150% increased impact force without increasing jump height (Peng et al., 2017); 35 cm in elite adolescent handball players, with no difference between 35 and 50 cm (Prieske et al., 2019); 60 cm after six weeks in collegiate Sanda athletes (Li et al., 2023); 40–60 cm for explosive strength but 60–80 cm for change-of-direction performance in elite female wrestlers (Bai et al., 2024); and 75–125% of individual squat-jump height in prepubescent boys (Bassa et al., 2022). These are five local calibration rules expressed in three non-equivalent units.

Drop-height prescriptions are also calibrated locally and expressed in non-equivalent units. In Division III athletes, heights below 75% of maximum vertical-jump height were favored, whereas 150% increased impact force without increasing jump height (Peng et al., 2017). In elite female wrestlers, 40–60 cm favored explosive strength and 60–80 cm favored change-of-direction performance within the same cohort (Bai et al., 2024). Prepubescent boys required heights scaled to 75–125% of their own squat-jump performance (Bassa et al., 2022). These results provide population- and outcome-specific calibration rules, not a transferable intensity scale. They also contrast with the individualized criterion in the source account discussed earlier in this review.

Drop height also moves the contact-time classifier itself. During bilateral drop jumps from 0.2, 0.4 and 0.6 m, force and time variables increased together, contact time was shorter at lower heights, and contact times for all jumps were reported as below 0.26 s (Ball et al., 2010). Increasing the box therefore lengthened contact and moved the exercise towards the slow category, even though greater height is often intended to increase loading. This does not prove that contact time and intensity are inversely related, because the reported contact times clustered near the threshold and did not form distinct categories. It does show that the prescription axis and the classification axis are not automatically aligned, as the impulse analysis in Section 5.2 predicts: a higher box raises the impulse to be absorbed and generated, and contact lengthens unless force rises in proportion. In the source chapter, an optimum contact duration rather than a one-sided ceiling was described (Schmidtbleicher, 1992).

### 7.2 Performance training: drills, hurdles and sprint contacts

In performance training the boundary is used to select drills that are expected to transfer to sprinting and jumping, and the relevant comparison is between the contact times of the drills and those of the target action. At maximal velocity, ground contact in 26 elite male sprinters lasted 96 ± 7 ms, with a range of 85–110 ms (Mattes et al., 2021), and the drills used to train fast SSC function seldom approach these values. Bilateral hurdle jumps over heights of 100–160% of countermovement-jump height produced contact times of approximately 178–209 ms, whereas unilateral hurdle jumps over 70–90% of that height produced 256–262 ms, and the countermovement jump itself produced approximately 810 ms (Cappa and Behm, 2011). Drop jumps from 0.2–0.6 m produced contact times below 0.26 s that lengthened with height (Ball et al., 2010). Contact in these drills lasts roughly twice to nearly three times as long as a maximal-velocity sprint contact, and a hurdle hop can fall on either side of the boundary depending on whether it is performed on one leg or two. The 250 ms line thus groups sprint contacts with bilateral hurdle jumps and low-box drop jumps while separating them from a unilateral hurdle hop. Yet the unilateral version imposes the required impulse on one limb and is closer in structure to a running contact. Applied to drill selection, the boundary tracks the wrong variable: transfer depends on whether a drill reproduces the impulse, loading rate and limb configuration of the target action, and those properties are expressed continuously (Section 5.2).

SSC category and ground contact time are rarely identified in practice surveys as primary reasons for exercise selection. Sprint coaches emphasized expected adaptation, practicality and target muscle groups, and 93% reported using hurdle jumps (Healy et al., 2021). Olympic sprint and jump coaches have described their programming in detail (Loturco et al., 2023), while elite Brazilian soccer practitioners also emphasized congested schedules, travel and regional constraints (Loturco et al., 2022). Plyometric training was reported by 95% of surveyed elite rugby union practitioners (Jones et al., 2016). The fast–slow distinction remains explicit in textbooks, research designs and applied guidance (Flanagan and Comyns, 2008), but it is not prominent in the decision criteria that practitioners report. Absence from survey responses does not show that the category is ignored; it indicates that its independent contribution to drill selection has not been demonstrated.

### 7.3 Rehabilitation: an injury-dependent progression gate

In rehabilitation the boundary performs a different function. It is used less to rank drills than to decide when a healing tissue may be exposed to reactive loading, and its meaning depends on the injury. After anterior cruciate ligament reconstruction, plyometric progression is organized along three axes (Buckthorpe and Della Villa, 2021). Ground reaction force ranges from about 1.5 to 7 times body mass across tasks; tasks move from bilateral to unilateral and from linear to multiplanar; and ground contact time is shortened progressively from 1–2 s in early stages towards 0.25–0.4 s and then below 200 ms for linear speed. Here the boundary is a late gate on the capacity of the knee to accept force at a high rate, and it is reached last. Soccer players about nine months after reconstruction, who had completed a comprehensive rehabilitation program, still showed lower and more asymmetrical vertical hop performance and reactive strength index than matched controls (Read et al., 2023). One explanation offered is that individuals lengthen ground contact to produce the force needed for propulsion and so compensate for reduced SSC function (Read et al., 2023). For tendinopathy the emphasis shifts to the tissue that stores and returns energy during short contacts. In the patellar tendon, a leg press at three times body weight and the landing phase of a vertical jump impose similar peak tendon forces, 5.2 and 5.17 ± 0.86 body weights, but loading rates of about 2 and 38.06 ± 11.55 body weights per second (Malliaras et al., 2015). A stop-land/jump sequence reaches 93 ± 23 body weights per second (Malliaras et al., 2015). The major change across these activities is the rate of loading, and loading is progressed from isometric and isotonic exercise to energy-storage exercise before return to sport (Malliaras et al., 2015). For the Achilles tendon, loading rate rises from 2.7–3.6 body weights per second during seated heel raises to 67.7 ± 25.9 body weights per second during single-leg lateral hopping (Baxter et al., 2021). Peak load rises from 3.3 ± 0.3 body weights in walking to 5.2 ± 0.9 in running and 7.3 in single-leg hopping (Baxter et al., 2021). In conservative management, tendon load is increased by increasing the speed of movement, and quick-rebounding heel rises are introduced to build tolerance to fast loads before plyometric exercise (Silbernagel et al., 2020). Plyometrics begin in a rebuilding phase from about week 3 to 12 under a pain-monitoring model (Silbernagel et al., 2020). In both cases short contacts come last, but for different reasons. After ligament reconstruction the limiting factor is the capacity of the knee and its musculature to accept force at a high rate; in tendinopathy it is the tolerance of the tendon itself to the loading rate at which energy is stored and returned. The same contact-time gate therefore certifies different capacities in different injuries.

In both settings the boundary indexes loading rate and its distribution between tissues (Section 5), not a category of exercise. Specifying the loading rate and peak force, as the tendon-loading data allow, is more informative than specifying the side of the line. Rehabilitation is also where the athlete-dependence of category membership matters most. A longer tendon (Ünver et al., 2024), a lower leg stiffness, or the compensatory lengthening of contact described after reconstruction (Read et al., 2023) all move contact time for a given task. A patient may therefore satisfy or fail a contact-time criterion for reasons unrelated to the tissue capacity that the criterion is meant to certify. A contact-time gate in rehabilitation should state which tissue it protects, which loading rate it corresponds to, and how the athlete’s own contact time under the task was measured.

### 7.4 Coupling the index used to calibrate and judge training

Reactive strength indices are used to select drop height, discriminate among exercises and evaluate performance. Single trials of the reactive strength index were reliable enough to support efficient individual drop-height testing, whereas time to stabilization was not (Flanagan et al., 2008). In the corresponding field protocol, an individual zone is defined as the height beyond which contact lengthens and the index falls (Flanagan and Comyns, 2008), and interday reliability of both the index and the derived optimal height was subsequently confirmed (Byrne et al., 2017). The modified index discriminated among five plyometric exercises (Ebben and Petushek, 2010), and contact time and the index correlated with competition performance in a small sample of trained weightlifters (Anastasiou et al., 2023). However, the reactive strength index and its modified form shared only 22% of their variance and differed by a standardized effect of 2.57 (McMahon et al., 2021), and other measures of SSC utilization also diverge (Suchomel et al., 2016). Because these indices include a time component, using them both to calibrate the system and to judge its success cannot provide an independent validation of the contact-time classification. The same coupling appears in rehabilitation, where the index is both a progression criterion and an outcome (Read et al., 2023).

Changing the basis of the classification would not remove plyometric drills, volume progression or exercise variety. Pooled evidence has linked larger gains to programs exceeding ten weeks, 20 sessions and 50 jumps per session, and has favored combinations of squat jumps, countermovement jumps and drop jumps over a single exercise form (Sáez de Villarreal et al., 2009). Contact time can remain useful because it covaries with relevant task properties. The unresolved issue is attribution: when intensity criteria disagree, which should determine progression, and when an index contributes both to classification and evaluation, what result would falsify the classification? These questions are converted into reporting and study-design requirements in **Table 2**.

**Table 2:** Reporting and study-design requirements for testing the fast–slow classification.

What should be reported or tested	By whom	Question made answerable
The drop height actually achieved, rather than the height of the platform	Primary studies	Whether the stimulus described is the stimulus delivered, and whether drop heights are comparable between studies (Sects. 7.1 and 9.1)
The instrument used to record contact time, together with its offset against a force plate	Primary studies	Whether contact times recorded in different laboratories sit on one scale (Sect. 9.1)
The criterion actually applied when an exercise is assigned to a category, rather than the label attached to it	Primary studies	Whether two studies describing fast SSC training describe the same intervention (Sects. 7.2 and 8)
Stratification by the criterion applied rather than by the label carried	Syntheses	Whether fast and slow SSC training differ from one another, a contrast the pooled literature is not currently arranged to form (Sect. 8)
Which of the parallel intensity criteria a progression follows, stated in advance	Study designers and practitioners	Which ranking a progression should follow when the criteria disagree (Sects. 5.2, 7.1 and 7.4)
The outcome by which a category is judged, reported separately from the index that defines it	Primary studies	What result would count as disconfirming for the classification (Sect. 7.4)
The impulse and the peak force or loading rate accompanying each reported contact time	Primary studies	Whether two drills with the same contact time impose the same mechanical demand, and whether a fast label reflects loading rate or only duration (Sects. 5.2 and 7.3)
Contact time, tendon geometry, task conditions and training response measured in the same participants over a sufficient intervention period	One prospective study	Whether category membership predicts training response after anatomy and context are accounted for (Sects. 6 and 9.2)
Whether force–time curve categories predict differential training response	One prospective study	Whether profile-based classification improves prescription enough to justify its measurement burden (Sects. 9.1 and 9.2)

## 8 Consequences for evidence synthesis

Pooled evidence has repeatedly shown that plyometric training improves performance across populations. Whether training classified as fast or slow produces different responses has not been established, because the included studies are generally not organized by those categories. This is a design limitation rather than evidence against the classification: a synthesis can contrast only the groups defined and reported by its constituent studies. No effect size is recalculated here and no published synthesis is challenged. The narrower question is whether the current evidence base can test the predictive value of the fast–slow distinction.

In the syntheses examined here, evidence is stratified by outcome, training mode, adaptation level or surface rather than by the criterion used to assign SSC type. In an early meta-analysis of 26 studies, jump-height gains were reported separately for the squat jump, countermovement jump, countermovement jump with arm swing and drop jump: 4.7%, 8.7%, 7.5% and 4.7%, respectively (Markovic, 2007). This outcome-based structure cannot compare fast- and slow-classified interventions; the drop jump is pooled as an outcome test rather than isolated as an intervention category. In later syntheses, plyometrics were compared with weightlifting, traditional resistance training or combined programs (Morris et al., 2022; Oliver et al., 2024), and adaptations were organized by physiological level and training surface (Markovic and Mikulic, 2010). In primary studies, interventions were often described by mode and duration without assigning drills to an SSC category (Spurrs et al., 2003). These choices are reasonable for their stated questions but leave the threshold untested. Nor have program variables yielded a stable moderator pattern at the pooled level. In a synthesis of 61 articles and 2,576 participants, analyzed training variables did not explain reactive strength index effects in meta-regression (p = 0.714–0.984; R² = 0), although categorical subgroup analyses suggested larger gains in adults and in longer or more frequent programs (Ramirez-Campillo et al., 2023). Six moderators in volleyball players produced no significant subgroup differences (Ramirez-Campillo et al., 2020), and analyses in female soccer players returned p values of 0.12–0.88 (Pardos-Mainer et al., 2021). Benefits in basketball players were reported across sex, age and program characteristics (Ramirez-Campillo et al., 2022). The published studies have therefore not consistently explained between-study variation, which is not the same as showing that program variables are unimportant to individuals.

Dose recommendations also depend on the outcome being optimized. For vertical-jump height, pooled evidence favored programs longer than ten weeks, with more than 20 sessions and more than 50 jumps per session (Sáez de Villarreal et al., 2009). For sprint performance, a synthesis from the same group favored programs shorter than ten weeks, at least 15 sessions and more than 80 combined jumps per session (Sáez de Villarreal et al., 2012). Different outcomes can require different doses, but no single program ranking transfers automatically across outcomes. Transfer is further limited because primary studies commonly express intensity as locally calibrated drop height rather than as impulse, loading rate or a standardized stimulus. The evidential base is also less independent than the number of reviews may suggest: in an umbrella review of 29 meta-analyses, only five included control-group comparisons, whereas 24 relied on pre-to-post effect sizes (Kons et al., 2023). For the present argument, the central limitation is more specific: a variable that has not been used to stratify the evidence cannot be validated by that evidence, which differs from concluding that the classification is unsupported. Reactive strength index has been pooled as an outcome (Ramirez-Campillo et al., 2023), but that does not test its role, or that of contact time, as a classifier. In no synthesis examined here were nominally fast SSC interventions grouped by the criterion actually applied, so the overlap among those intervention sets is unknown, and such a synthesis first requires the operational criterion to be reported in primary studies (**Table 2**).

## 9 From a universal threshold to individual profiles?

Moving from a universal threshold to an individual profile is not the same as choosing a new cut-point. A profile records how an athlete responds across tasks and loads rather than forcing every observation into two classes. Force–time profile approaches already exist in youth research and have been applied in more than one cohort. They also recover an element of the source account, in which drop-height prescription was individualized. The trade-off is measurement burden: a threshold requires only a side-of-line decision, whereas a profile requires comparable values across trials, tasks and instruments. Evidence for that comparability is mixed, particularly for the short contacts assigned to the fast category.

### 9.1 Force–time profiles and their measurement demands

One approach classifies drop jumps from force–time curve shape, using the presence of an impact peak and a spring-like trace to identify poor, moderate or good SSC function. Players classified as good showed shorter ground contact, less center-of-mass displacement and greater take-off force than other groups, and they performed better on every measured drop-jump outcome except jump height; nevertheless, 9.9% of participants beyond peak height velocity were classified as poor (Pedley et al., 2022). The same scheme applied to a separate cohort classified 17.8% of post-peak-height-velocity athletes as poor, the figure reported earlier in the review (Kumar et al., 2024); the two proportions describe different samples and are not directly comparable. Here, contact time is a correlate rather than the definition. In a related comparison of unimodal and bimodal force profiles, shorter contact, greater leg stiffness and greater eccentric peak power were found in the unimodal group, while reactive strength index did not differ (Peng et al., 2024). Curve shape may therefore capture distinctions that a single time-based index misses, and it sits closer to the impulse-based description of Section 5.2, but its predictive value for training prescription has not been established.

A profile also requires contact time to be interpreted as task- and instrument-specific rather than as a stable athlete trait. Within the same participants, contact times in vertical and horizontal drop jumps correlated weakly (r = 0.222) and were not associated with vertical jump height (r = 0.152) or horizontal jump distance (r = 0.261), despite acceptable reliability within each task (Ball and Zanetti, 2012). Drop-jump contact time has also been identified as an exception to otherwise high reproducibility across strength and power measures (Washif et al., 2024). Instrument choice introduces systematic bias. An optical system with intraclass coefficients above 0.975 overestimated contact time by 0.005 ± 0.002 s because its units sat 0.003 m above the floor (Healy et al., 2016), and an inertial sensor differed from force-plate values across reported variables, with reliability improving as contacts lengthened (Montoro-Bombú et al., 2022). The delivered stimulus can also differ from the prescribed one: in one elite squad performing bounce drop jumps from a 20 cm platform, actual dropping heights ranged from 14.87 to 29.85 cm (Costley et al., 2018). Reliable estimation from foot- and lower-spine-mounted sensors is possible, with coefficients of variation below 9.9% (Horsley et al., 2021), but comparison requires the method to be reported and calibrated.

Profiles based on reactive strength indices inherit the differences among those indices. Bland–Altman comparison of the reactive strength index and its modified form produced biases of 0.50–0.57 and limits of agreement of 1.27–1.51, wider than mean scores of 0.97–1.05 and 0.42, with performance-dependent bias (Louder et al., 2021). The two indices have also been found unsuitable for interchange (McMahon et al., 2021). In one dataset, the modified index correlated more strongly with jump height (r = 0.75–0.87) than with time to take-off (r = 0.45–0.54) (Vieira and Tufano, 2021), suggesting that the height term may dominate its interpretation. A profile is therefore not automatically more valid than a threshold; its component measurements and intended decision must be specified.

### 9.2 A testable path forward

Most immediate improvements require transparent reporting rather than a new device. Four items matter: actual drop height rather than platform height; the contact-time instrument and its offset against a force plate; the operational criterion used to assign each exercise to a category; and, following Section 5.2, the impulse and the peak force or loading rate that accompanied the reported contact time. These details would allow nominally fast SSC interventions to be compared by what investigators actually did. The decisive prospective design would measure contact time, tendon geometry, task conditions and training response in the same participants over a sufficient intervention period, and could then test whether category membership predicts response beyond anatomy and context. Force–time classification requires a parallel test: it was developed to describe maturation-related function, not to prescribe training, and no prospective study has shown that its categories predict differential response. The reporting and design steps needed are set out in **Table 2**. A profile should replace a threshold only if it improves a defined decision enough to justify its additional measurement burden. Several questions raised here exceed what a critical review can settle and would justify a systematic review of their own. They include the contact times, impulses and loading rates actually recorded across the drills labelled fast SSC; the tendon–muscle partition of length change across the contact-time range in trained athletes rather than in single muscles; the contact-time criteria applied in rehabilitation protocols for different injuries; and the proportion of published fast SSC interventions in which the criterion was measured rather than assumed.

## 10 Conclusion

Current evidence does not validate 250 ms as an independent fast–slow SSC classifier. The original definition combined movement duration with joint displacement; contact time alone was often used in later work. No mechanistic discontinuity at 250 ms has been shown, and in the only direct validation study alternative cut-points were identified and Achilles tendon length was the strongest discriminator. What contact time does index is the distribution in time of a required impulse and, with it, the loading rate imposed on tissue and the partition of length change between tendon and muscle. These vary continuously with frequency, height, instruction and the tissue properties of the athlete. The boundary therefore has a different function in each applied setting. In performance training, transfer depends on reproducing the impulse and loading rate of the target action; in rehabilitation, the boundary gates the exposure of a healing tissue to reactive loading, and its meaning depends on the injury. These strands differ in strength as well as in kind. The provenance findings rest on documents a reader can compare directly, and the synthesis argument concerns how existing meta-analyses are organized. The mechanistic case for a breakpoint remains the least secure. The muscle–tendon and impulse evidence rests on direct measurement but comes from hopping and drop jumping in a few laboratory cohorts. The evidence on individual category membership rests principally on a single study of 46 recreationally active men. The strands converge in direction, but none of them alone would carry the conclusion, and several of the questions they raise, listed in Section 9.2, warrant a systematic review of their own. This neither invalidates the threshold universally nor supports a replacement. Until category membership predicts training response beyond anatomy, task and measurement, contact time should remain a descriptive variable rather than a prescription rule.
